# Supplementary material for: Tierra Del Fuego: What Is Left from the Precolonial Male Lineages?
Source: Genes (Basel). 2022 Sep 23;13(10):1712. doi: 10.3390/genes13101712 (PMC9601523; doi:10.3390/genes13101712)
Supplement: Supplementary file 1 [file genes-13-01712-s001.zip › Suplementary Figures.pdf]

SUPPLEMENTARY FIGURES

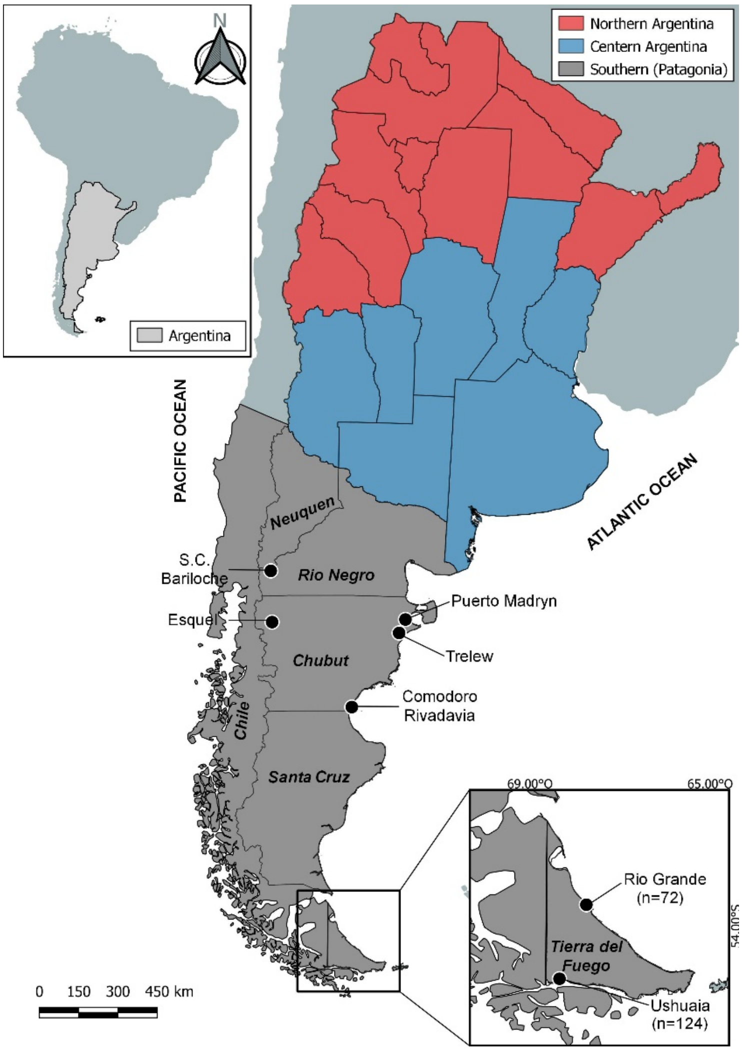

Supplementary Figure S1. Map of Argentina and the Southern tip of the American continent. The cities where the sample collections took place – Río Grande and Ushuaia - are marked in the map indicating the number of individuals participating in this study for each city.

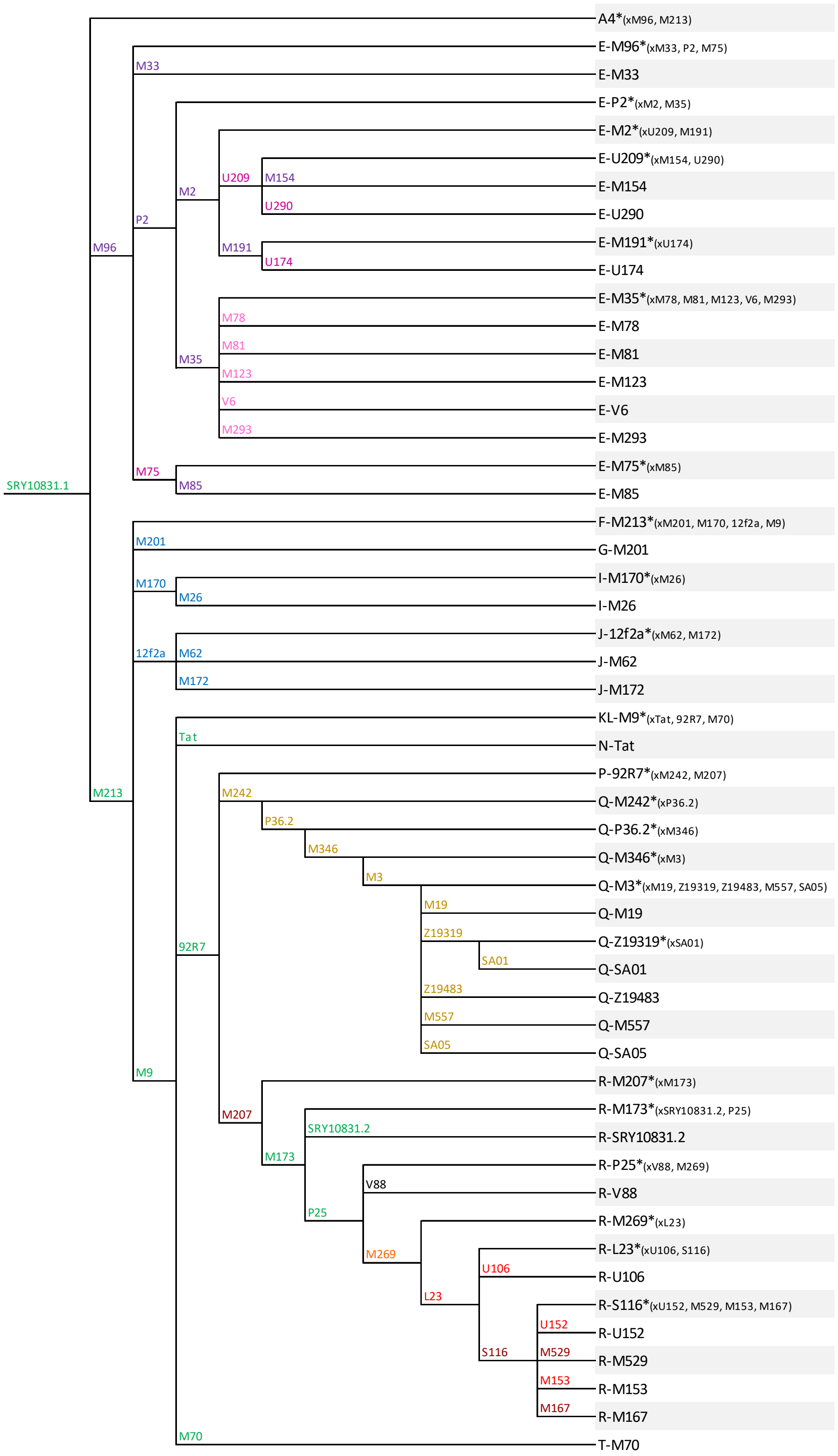

Supplementary Figure S2. Phylogenetic tree of the Y-SNPs genotyped in this study, and corresponding haplogroups. The markers are represented in different colors based on the multiplex in which they are embedded (green – Mx 1; dark pink – Mx E1; pink - Mx E2; purple – both Mx E1 and E2; blue – Mx 2; golden – Mx Q; red – Mx R1; orange – Mx R2; dark red – both Mx R1 and R2; black - singleplex).

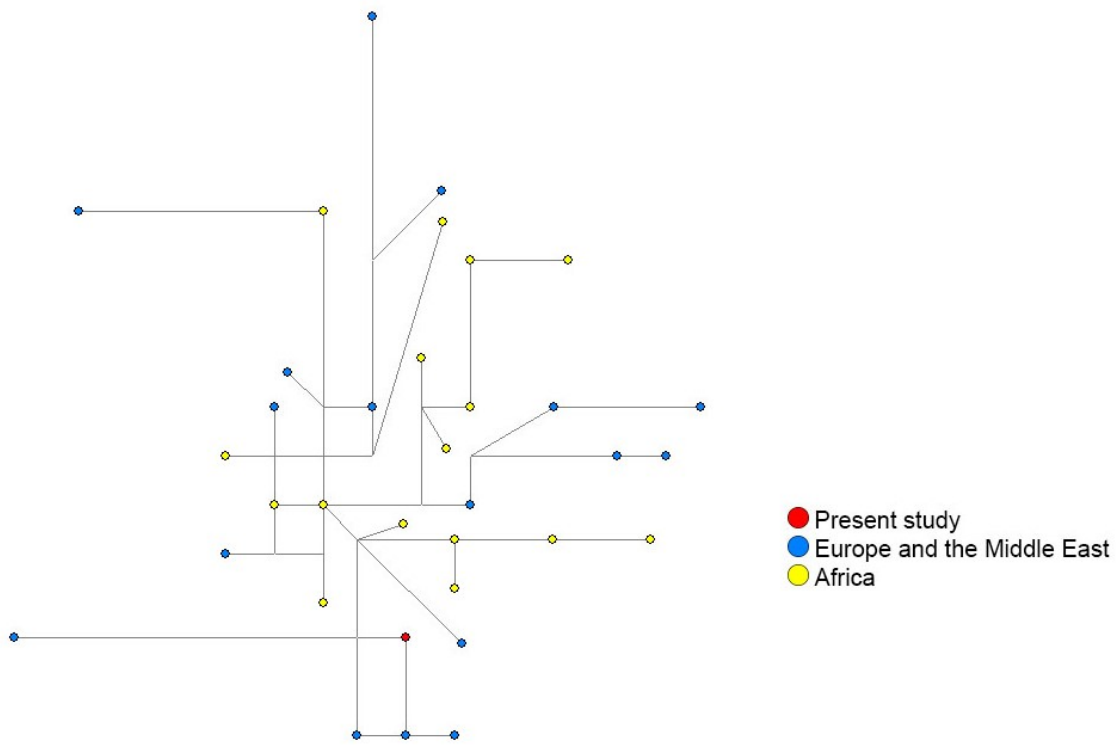

Supplementary Figure S3. E-M35* median-joining network with 10 Y-STRs (DYS19, DYS389I, DYS389II, DYS390, DYS391, DYS392, DYS393, DYS437, DYS438, and DYS439) common to all populations. In the network are included 1 sample from our dataset, 17 Eurasian (Adams et al. 2008; Boattini et al. 2013; Rębała et al. 2013; Zalloua et al. 2008), and 16 African (De Filippo et al. 2011; Larmuseau et al. 2015; Rosa et al. 2007).

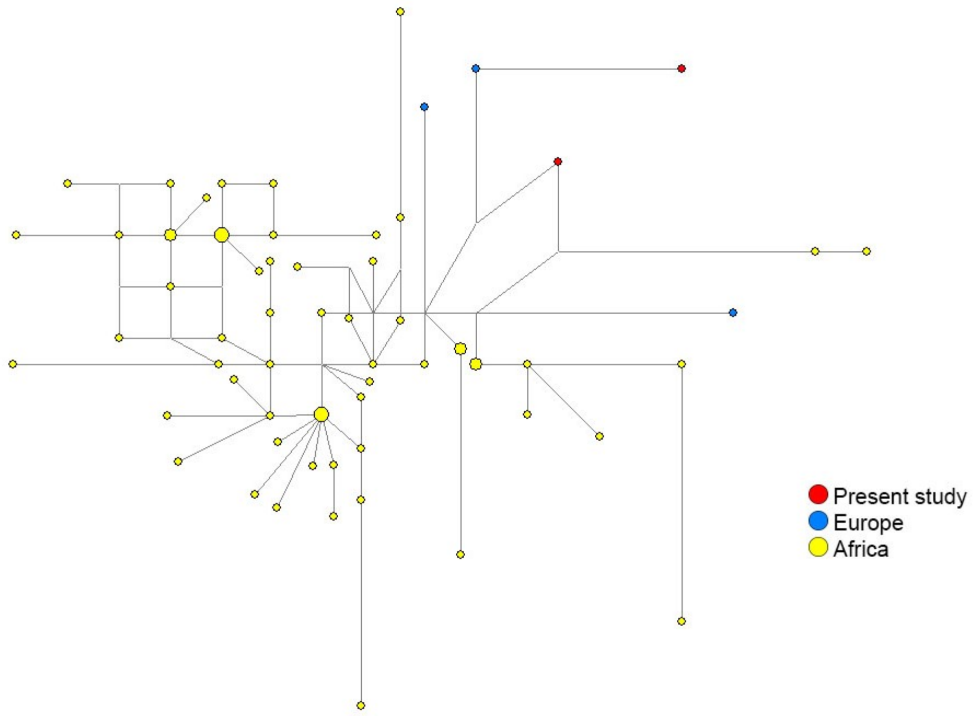

Supplementary Figure S4. R-V88 median-joining network with 12 Y-STRs (DYS19, DYS385a, DYS385b, DYS389I, DYS389II, DYS390, DYS391, DYS392, DYS393, DYS437, DYS438, and DYS439) common to all populations. In the network are included 2 samples from our dataset, 3 European (Boattini et al. 2013; Di Cristofaro et al. 2018), and 62 African (Berniell-Lee et al. 2009; Di Filippo et al. 2011; Fortes-Lima et al. 2015; González et al. 2013; Larmuseau et al. 2015; Rosa et al. 2007).

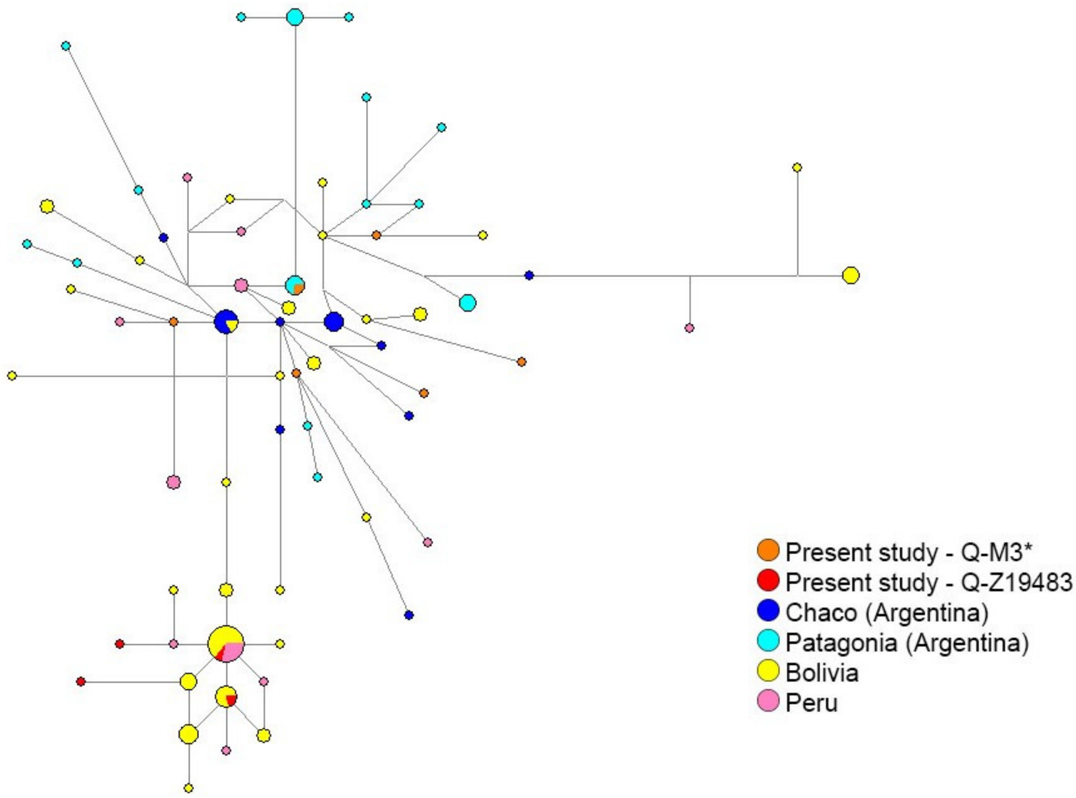

Supplementary Figure S5. Haplogroup Q median-joining network with 10 Y-STRs (DYS19, DYS389I, DYS389II, DYS390, DYS391, DYS392, DYS393, DYS437, DYS438, and DYS439) common to all populations. A total of 115 samples were included in the network: 6 belonging to Q-M3* and 4 belonging to Q-Z19483 from our dataset, 52 natives from Bolivia (all samples Q-M3) (Gayà-Vidal et al. 2011), 16 natives from Peru [7 samples belonging to the haplogroup Q-M3 and 9 to the haplogroup Q-M3*(xM19)] (Barbieri et al. 2017; Roewer et al. 2013), 16 natives from Chaco, Argentina, and 21 natives from Patagonia, Argentina [all samples Q-M3*(xM19)] (Roewer et al. 2013).

References:

Adams, S. M., Bosch, E., Balaresque, P. L., Ballereau, S. J., Lee, A. C., Arroyo, E., López-Parra, A. M., Aler, M., Grifo, M. S., Brion, M., Carracedo, A., Lavinha, J., Martínez-Jarreta, B., Quintana-Murci, L., Picornell, A., Ramon, M., Skorecki, K., Behar, D. M., Calafell, F., & Jobling, M. A. (2008). The genetic legacy of religious diversity and intolerance: paternal lineages of Christians, Jews, and Muslims in the Iberian Peninsula. *American Journal of Human Genetics, 83*(6), 725–736.

Barbieri, C., Sandoval, J. R., Valqui, J., Shimelman, A., Ziemendorff, S., Schröder, R., Geppert, M., Roewer, L., Gray, R., Stoneking, M., Fujita, R., & Heggarty, P. (2017). Enclaves of genetic diversity resisted Inca impacts on population history. *Scientific Reports, 7*(1), 17411.

Berniell-Lee, G., Calafell, F., Bosch, E., Heyer, E., Sica, L., Mouguiama-Daouda, P., van der Veen, L., Hombert, J. M., Quintana-Murci, L., & Comas, D. (2009). Genetic and demographic implications of the Bantu expansion: insights from human paternal lineages. *Molecular Biology and Evolution, 26*(7), 1581–1589.

Boattini, A., Martinez-Cruz, B., Sarno, S., Harmant, C., Useli, A., Sanz, P., Yang-Yao, D., Manry, J., Ciani, G., Luiselli, D., Quintana-Murci, L., Comas, D., Pettener, D., & Genographic Consortium (2013). Uniparental markers in Italy reveal a sex-biased genetic structure and different historical strata. *PLoS ONE, 8*(5), e65441.

De Filippo, C., Barbieri, C., Whitten, M., Mpoloka, S. W., Gunnarsdóttir, E. D., Bostoen, K., Nyambe, T., Beyer, K., Schreiber, H., de Knijff, P., Luiselli, D., Stoneking, M., & Pakendorf, B. (2011). Y-chromosomal variation in sub-Saharan Africa: insights into the history of Niger-Congo groups. *Molecular Biology and Evolution, 28*(3), 1255–1269.

Di Cristofaro, J., Mazières, S., Tous, A., Di Gaetano, C., Lin, A. A., Nebbia, P., Piazza, A., King, R. J., Underhill, P., & Chiaroni, J. (2018). Prehistoric migrations through the Mediterranean basin shaped Corsican Y-chromosome diversity. *PloS ONE*, *13*(8), e0200641.

Fortes-Lima, C., Brucato, N., Croze, M., Bellis, G., Schiavinato, S., Massougbodji, A., Migot-Nabias, F., & Dugoujon, J. M. (2015). Genetic population study of Y-chromosome markers in Benin and Ivory Coast ethnic groups. *Forensic Science International: Genetics, 19*, 232–237.

Gayà-Vidal, M., Moral, P., Saenz-Ruales, N., Gerbault, P., Tonasso, L., Villena, M., Vasquez, R., Bravi, C. M., & Dugoujon, J. M. (2011). mtDNA and Y-chromosome diversity in Aymaras and Quechuas from Bolivia: different stories and special genetic traits of the Andean Altiplano populations. *American Journal of Physical Anthropology, 145*(2), 215–230.

González, M., Gomes, V., López-Parra, A. M., Amorim, A., Carracedo, A., Sánchez-Diz, P., Arroyo-Pardo, E., & Gusmão, L. (2013). The genetic landscape of Equatorial Guinea and the origin and migration routes of the Y chromosome haplogroup RV88. *European Journal of Human Genetics: EJHG, 21*(3), 324–331.

Larmuseau, M. H., Vessi, A., Jobling, M. A., van Geystelen, A., Primativo, G., Biondi, G., Martínez-Labarga, C., Ottoni, C., Decorte, R., & Rickards, O. (2015). The Paternal Landscape along the Bight of Benin - Testing Regional Representativeness of West-African Population Samples Using Y-Chromosomal Markers. *PLoS ONE, 10*(11), e0141510.

Rębała, K., Martínez-Cruz, B., Tönjes, A., Kovacs, P., Stumvoll, M., Lindner, I., Büttner, A., Wichmann, H. E., Siváková, D., Soták, M., Quintana-Murci, L., Szczerkowska, Z., Comas, D., & Genographic Consortium (2013). Contemporary paternal genetic landscape of Polish and German populations: from early medieval Slavic expansion to post-World War II resettlements. *European Journal of Human Genetics: EJHG, 21*(4), 415–422.

Roewer, L., Nothnagel, M., Gusmão, L., Gomes, V., González, M., Corach, D., Sala, A., Alechine, E., Palha, T., Santos, N., Ribeiro-Dos-Santos, A., Geppert, M., Willuweit, S., Nagy, M., Zweynert, S., Baeta, M., Núñez, C., Martínez-Jarreta, B., González- Andrade, F., Fagundes de Carvalho, E., Aparecida da Silva, D., Builes, J. J., Turbón, D., Lopez Parra, A. M., Arroyo-Pardo, E., Toscanini, U., Borjas, L., Barletta, C., Ewart, E., Santos, S., & Krawczak, M. (2013). Continent-wide decoupling of Y-chromosomal genetic variation from language and geography in native South Americans. *PLoS Genetics, 9*(4), e1003460.

Rosa, A., Ornelas, C., Jobling, M. A., Brehm, A., & Villems, R. (2007). Y-chromosomal diversity in the population of Guinea-Bissau: a multiethnic perspective. *BMC Evolutionary Biology*, *7*, 124.

Zalloua, P. A., Xue, Y., Khalife, J., Makhoul, N., Debiane, L., Platt, D. E., Royyuru, A. K., Herrera, R. J., Hernanz, D. F., Blue-Smith, J., Wells, R. S., Comas, D., Bertranpetit, J., Tyler-Smith, C., & Genographic Consortium (2008). Y-chromosomal diversity in Lebanon is structured by recent historical events. *American Journal of Human Genetics, 82*(4), 873–882.
