# Supplementary material for: Tierra Del Fuego: What Is Left from the Precolonial Male Lineages?
Source: Genes (Basel). 2022 Sep 23;13(10):1712. doi: 10.3390/genes13101712 (PMC9601523; doi:10.3390/genes13101712)
Supplement: Supplementary file 1 [file genes-13-01712-s001.zip › Supplementary Information.pdf]

SUPPLEMENTARY INFORMATION

Paternal genealogical data

1. Known paternal geographic origin

Out of 196 participants in the present study, seven individuals did not provide information about their paternal geographic origin. In 89 cases, we obtained information on the birthplace of the three preceding generations following the paternal lineage. For 65 individuals, we could only retrieve information of the two preceding generations, and for 32 individuals, we only have the birthplace of the father. Additionally, two individuals lacked information just on the birthplace of the grandfather, and one individual on the birthplace of the father.

For 46.4% of the donors, the known paternal origin preceded from different Argentine provinces (Supplementary Table SI1), mostly from central Argentina, a region from several Argentines migrated to Tierra del Fuego, according to the latest census data. Individuals with a known European paternal heritage comprise 36.2% of the entire sample. This share is divided mainly between Italy (14.8%) and Spain (12.8%), which is consistent with the historical records on European flows to Argentina. The rest of Europe accounts for 8.7%, which includes France, Germany, Poland, Portugal, Ireland, Austria, Czechia, and Russia. Additionally, 3.1% of the individuals declared a Middle Eastern paternal ancestry. Only 10.7% of the donors indicated a Latin American origin, of which slightly more than half were from Chile (5.6%), followed by Bolivia (2.0%) and Paraguay (1.5%). As addressed in the introduction, these three countries bordering Argentina have been the main ones from where the immigrants in Tierra del Fuego have moved out in the recent decades.

Supplementary Table S1. Birthplaces of the oldest known paternal ancestor (great-grandfather, grandfather, or father) and their relative and absolute frequencies. Furthermore, it is depicted the absolute frequencies of the three haplogroup continental origins [Eurasian (EUR), Native American (NAM), and African (AFR)].

Region	%	n		%	Total (n)	EUR (n)	NAM (n)	AFR (n)
Argentina	46.4%	91	Northern Argentina	10.2%	**20**	17	3	-
			Central Argentina	34.2%	**67**	65	2	-
			Southern Argentina/Patagonia	2.0%	**4**	3	1	-
Europe and the Middle East	39.3%	77	Italy	14.8%	**29**	29	-	-
			Spain	12.8%	**25**	25	-	-
			Rest of Europe	8.7%	**17**	17	-	-
			Middle East	3.0%	**6**	6	-	-
Latin America	10.7%	21	Chile	5.6%	**11**	11	-	-
			Bolivia	2.0%	**4**	2	2	-
			Paraguay	1.5%	**3**	2	-	1
			Rest of Latin America	1.5%	**3**	3	-	-
Unknown	3.6%	7	-	3.6%	**7**	5	2	-

2. Generational differences

For those individuals who provided paternal origins up to great-grandfathers, it is noticeable a predominance of European and Middle Eastern origins of the great-grandfathers (Supplementary Figure SI1). The European and Middle Eastern provenances decrease from generation to generation. Most of the grandfathers and fathers were born in Argentina, contrasting markedly with the provenance of the great-grandfathers.

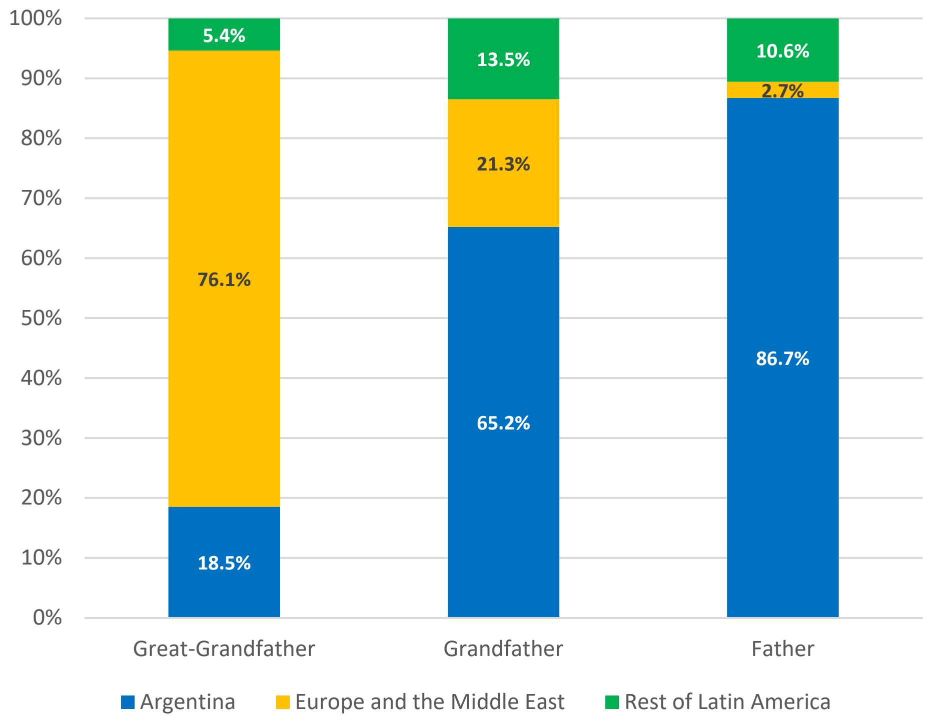

Supplementary Figure S1. Birthplace frequencies of the donors' preceding generations - Great-grandfathers (n=92), grandfathers (n=155), and fathers (n=188) - divided into three categories (Argentina, rest of Latin America, and Europe and the Middle East).

2. Birthplace of the donors

As illustrated in Supplementary Figure SI2, out of the 196 participants in this study, 185 stated Argentina as their birthplace. The geographical distribution of these Argentine-born individuals is largely congregated in central Argentina, mainly in the provinces of Buenos Aires (n=68), Cordoba (n=14), and Santa Fe (n=13). Southern Argentina/Patagonia is the next, most of the individuals being born in the Tierra del Fuego province (n=43). The other Patagonian individuals were born in Santa Cruz and Chubut provinces (both provinces n=3). Having less representation, those born in northern Argentina come from a variety of provinces, such as Salta (n=6), Tucuman, Misiones, Corrientes, and Chaco (n=4).

Only eight individuals in this study were born in other Latin American countries, four Chilean and the others from Bolivia, Brazil, Mexico, and Venezuela. Finally, one of

the donors reported to be from Spain and is consequently the only donor born outside the American continent in our sample.

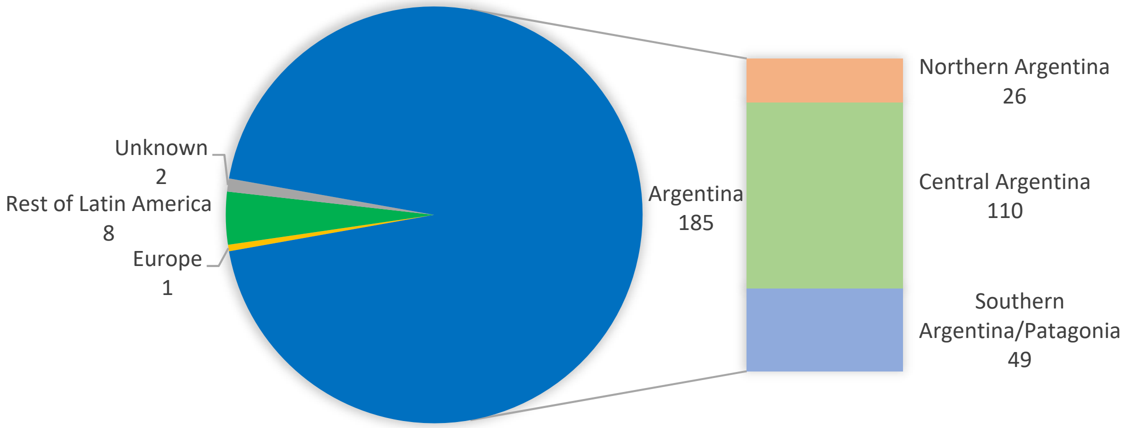

Supplementary Figure S2: Distribution of the birthplaces of the sample donors separated into three categories (Argentina, Rest of Latin America, and Europe). The Argentine category is subdivided into three groups (northern Argentina, central Argentina, and southern Argentina/Patagonia). Under each geographic location is the corresponding number of individuals sampled.
